# An Innovative “Tooth‐On‐Chip” Microfluidic Device Emulating the Structure and Physiology of the Dental Pulp Tissue

**DOI:** 10.1002/adhm.202502080

**Published:** 2025-08-21

**Authors:** Alessandro Cordiale, Deborah Stanco, Roberta Visone, Bernd Stadlinger, Pierfrancesco Pagella, Marco Rasponi, Thimios A. Mitsiadis

**Affiliations:** ^1^ Institute of Oral Biology Centre of Dental Medicine Medical Faculty University of Zurich Zurich 8032 Switzerland; ^2^ Department of Electronics Information and Bioengineering Politecnico di Milano Milano 20133 Italy; ^3^ Clinic of Cranio‐Maxillofacial and Oral Surgery Centre of Dental Medicine Medical Faculty University of Zurich Zurich 8032 Switzerland; ^4^ Laboratory of Molecular Materials Division of Biophysics and Bioengineering Department of Physics Chemistry and Biology (IFM) Linköping University Linköping 58183 Sweden; ^5^ Foundation for Research and Technology-Hellas (FORTH) University of Crete Heraklion 70013 Greece

**Keywords:** “tooth‐on‐chip”, dental pulp, dentine, endothelial cells, innervation, mesenchymal stem cells, microfluidics, odontoblasts, tooth, trigeminal ganglion, vasculature

## Abstract

The dental pulp is a highly vascularized and innervated connective tissue composed of various cell types, including fibroblasts, odontoblasts, mesenchymal stem cells, neuronal, and endothelial cells. The interplay between these diverse cell populations is pivotal for dental pulp tissue homeostasis and regeneration after carious infections and traumatic tooth lesions. Despite the great clinical need, comprehensive in vitro models that accurately recapitulate the complexity of the dental pulp are still missing, hampering the development of novel, faster, and more effective therapies. In this study, an innovative “tooth‐on‐chip” microfluidic device is presented to emulate the composition and three‐dimensional structure of the dental pulp tissue in vitro. Co‐culture of human dental pulp stem cells, odontoblast‐like cells, endothelial cells, and trigeminal neurones in this miniaturized system successfully reproduced the structural organization and physiology of the dental pulp. The microfluidic device integrated various compartments that allowed the generation of complex vascular and neuronal networks, the formation of stem cell perivascular niches, and the formation of an odontoblast/dentine interface. The “tooth‐on‐chip” device represents a conceptual leap in replicating dental pulp physiology in vitro, offering a state‐of‐the‐art platform to study dental pulp physiology and pathology and serving as a benchmark to create more advanced tooth simulation systems.

## Graphical abstract

This work presents a “tooth‐on‐chip” device that mimics dental pulp tissue. By co‐culturing key cell types, it recreates vascular networks, stem cell niches, the odontoblast/dentine interface, and trigeminal innervation. This innovative platform provides a unique model of dental pulp structure and physiology, with significant potential for accelerating the development of faster, more effective treatments for dental diseases.

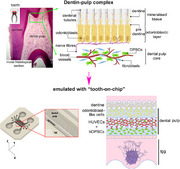

## 1 Introduction

**Figure 1:**
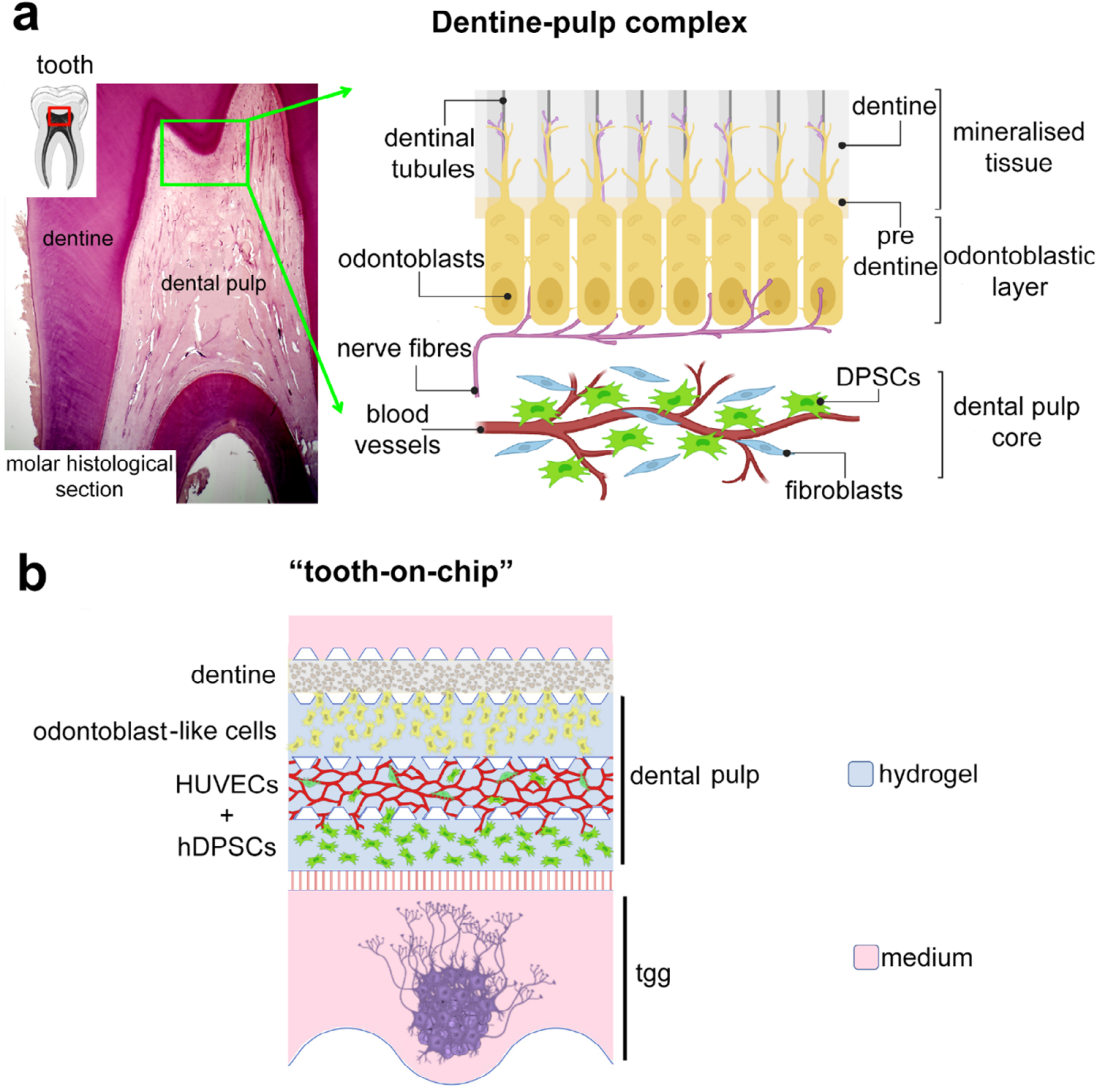
Dental microanatomy and microfluidic model. a) Left: haematoxylin‐eosin staining of a human molar histological section showing the basic organisation of the human dental pulp. Right: schematic representation of the dentine‐pulp interface. b) Tooth‐on‐chip model: schematic representation of the chip after the cell seeding: the dental pulp compartment consisted of the dentine layer followed by odontoblasts, HUVECs and hDPSCs populations, and the trigeminal ganglion compartment hosted trigeminal ganglion derived from mouse embryos.

Teeth are composed of hard and soft tissues (**Figure** **1**a). Enamel, produced by the epithelium‐derived ameloblasts, is the highest mineralized biological tissue in nature and covers the tooth crown. It is supported by dentine, a less mineralized hard tissue produced by the mesenchyme‐derived odontoblasts. These two dental hard tissues protect the highly vascularized and innervated connective dental pulp tissue at the internal central part of the teeth. Blood vessels are essential components of perivascular stem cell niches where various stem cell populations reside.^[^ ^1^, ^2^, ^3^, ^4^, ^5^ ^]^ Human dental pulp stem cells (hDPSCs) possess the self‐renewal ability and multilineage differentiation potential, which ensure pulp homeostasis and regeneration,^[^ ^6^, ^7^, ^8^, ^9^ ^]^ and can be used in dental clinics, either alone or in combination with innovative bioengineering products.^[^ ^10^, ^11^, ^12^ ^]^

Carious and traumatic dental lesions often damage the dental pulp tissue by inducing inflammation and necrosis.^[^ ^13^, ^14^ ^]^ Conventional dental treatments in these cases include the removal of the pathological dental pulp tissue, followed by filling the empty dental pulp chamber with materials that do not restore entire tooth functionality and physiology.^[^ ^15^, ^16^, ^17^, ^18^, ^19^, ^20^, ^21^ ^]^ Regenerative dentistry aims to regenerate physiological dental tissues, thus overcoming the limitations imposed by artificial dental materials.^[^ ^22^, ^23^ ^]^ This can be achieved by taking advantage of the regenerative potential of stem cells, combined with recent advances in genetics, tissue engineering, and nanotechnology.^[^ ^22^, ^24^ ^]^

Despite the encouraging results of the last two decades,^[^ ^25^, ^26^, ^27^, ^28^, ^29^, ^30^, ^31^, ^32^ ^]^ dental regenerative therapies are still not applicable in dental clinics. An integrated vision of dental pulp biology is missing, hampering the proper understanding of tooth physiology and pathology. This is tightly connected with the absence of appropriate experimental platforms to model and emulate the human dental pulp tissue in its entire complexity. In vivo experiments using various animal models do not entirely recapitulate human biology due to the intrinsic interspecies differences.^[^ ^33^, ^34^, ^35^ ^]^ Similarly, in vitro experiments based on 2D cell cultures are simplistic and often fail to mimic tissue biological complexity.^[^ ^36^, ^37^ ^]^ Therefore, there is a need to establish reliable preclinical models for understanding and recapitulating human tissue physiology and functionality and predicting therapeutic outcomes.

The “organ‐on‐chip” technology is applied to generate a variety of miniaturized tissues and organs, emulating their physiology and function.^[^ ^38^ ^]^ These 3D microfluidic systems use tuneable geometrical features and gels that mimic the complexity and physiopathology of specific tissues.^[^ ^38^ ^]^ 3D tissue‐specific microenvironments within these devices can be implemented to control cell‐cell and cell‐matrix interactions and biochemical and mechanical cues, all crucial elements for tissue development, remodeling, and regeneration.^[^ ^39^ ^]^ Furthermore, “organ‐on‐chip” systems are used to replicate the responses of human tissues to drugs and therapeutic agents.^[^ ^38^ ^]^ “Organ‐on‐chip” bioreactors comprise separate yet communicated compartments for the co‐culture of various cell populations, each in their optimized conditions, and channels that provide medium flow.^[^ ^38^, ^40^ ^]^ Optionally, these devices incorporate actuator components and biosensors for controlling or measuring the mechanical stress, metabolism, and electric potential.^[^ ^38^, ^40^ ^]^ “Organ‐on‐chip” devices have already been used to model, amongst others, the liver, intestine, lung, muscle, and heart and to mimic multi‐organ behavior and interactions.^[^ ^41^, ^42^, ^43^, ^44^, ^45^, ^46^, ^47^, ^48^, ^49^, ^50^, ^51^, ^52^ ^]^ In the first application of microfluidic co‐culture technologies to dental tissues, we showed that the co‐culture of trigeminal ganglia and tooth germs from different developmental stages recapitulates the dynamics of tooth innervation observed in vivo.^[^ ^53^ ^]^ We then exploited microfluidic co‐culture devices to assess the neurotrophic potential of several cell types, including dental pulp stem cells, bone marrow stromal cells,^[^ ^54^ ^]^ and cells isolated from human oral cancers.^[^ ^55^ ^]^ In the following years, custom microfluidic systems have been designed to emulate tooth‐specific tissues and features to unprecedented details. A particular microfluidic platform was developed to evaluate the effects of signalling molecules secreted from human gingival fibroblasts or periodontal ligament stem cells on the osteogenic commitment of human exfoliated deciduous tooth stem cells.^[^ ^56^ ^]^ A first microfluidic model of the dentin‐pulp interface was then proposed, allowing the monitoring of the interactions of apical dental papilla stem cells with dentine and dental restorative materials, such as resins.^[^ ^57^ ^]^ This device was then modified to culture hDPSCs in a 3D collagen matrix and to include a biofilm of *Streptococcus mutans* and was used to model the interaction of hDPSCs with calcium silicate cements.^[^ ^58^ ^]^ Other specific systems were then developed to investigate the interaction between vasculature and different populations of dental stem cells. These devices proved extremely valuable to determine the effect of stem cells from the apical papilla‐on vessel sprouting, and the role of semaphorin‐plexin signalling in the stabilization of vessels induced by perivascular stem cells from human exfoliated deciduous teeth.^[^ ^59^, ^60^ ^]^ A microfluidic chip was also designed to generate odontoblasts processes in vitro, by guiding their projection through 2 µm microchannels that mimic dentine tubules confinement.^[^ ^61^ ^]^

Besides pulp tissue, periodontal tissue has been the target of several microfluidics‐based emulation systems. Different “gingiva‐on‐chip” and oral mucosa models have been introduced to study host‐material and host‐microbiome interaction.^[^ ^62^, ^63^, ^64^, ^65^, ^66^, ^67^ ^]^ Among them, a model for gingival tissue inflammation was designed by using a commercially available high‐throughput platform. Such model enabled recirculation of the culture medium and recapitulated the barrier function of gingival epithelium, as measured through transepithelial resistance (TEER) sensors over time. TEER values showed a viable and physiological barrier that responded to inflammatory stimuli through a reduction in barrier tightness and secretion of specific inflammatory cytokines and chemokines.^[^ ^68^ ^]^ More recently, a “periodontal ligament‐on‐chip” device has been developed, which allowed the formation of a vascular network and the study of its interaction with the human periodontal ligament stem cells.^[^ ^69^ ^]^ While all these recently introduced “tooth‐on‐chip” devices are valuable and important for further understanding dental tissue biology, they do not recapitulate the complexity of dental tissues.^[^ ^70^, ^71^, ^72^, ^73^ ^]^ Indeed, despite the impressive advances in the field, a microfluidic system emulating the complexity of the entire dental pulp tissue, encompassing stem niches, vascularization, innervation, odontoblast‐like cells, and dentine, does not exist to date.

In the present study, we tackled this challenge and describe a novel “tooth‐on‐chip” device designed and developed to recapitulate the complexity and heterogeneity of the human dental pulp tissue and mimic its physiology. Our microfluidic system hosts hDPSCs, hDPSC‐derived odontoblast‐like cells, endothelial cells, trigeminal nerves, and dentine microparticles in multiple compartments (Figure 1b). It was technically and biologically validated in terms of mass transport, hydrogel composition, cell viability, proliferation and differentiation, and the formation of vascular and neuronal networks.

## 2 Results

### 2.1 Metabolites and Drug Molecules Mass Transport

**Figure 2:**
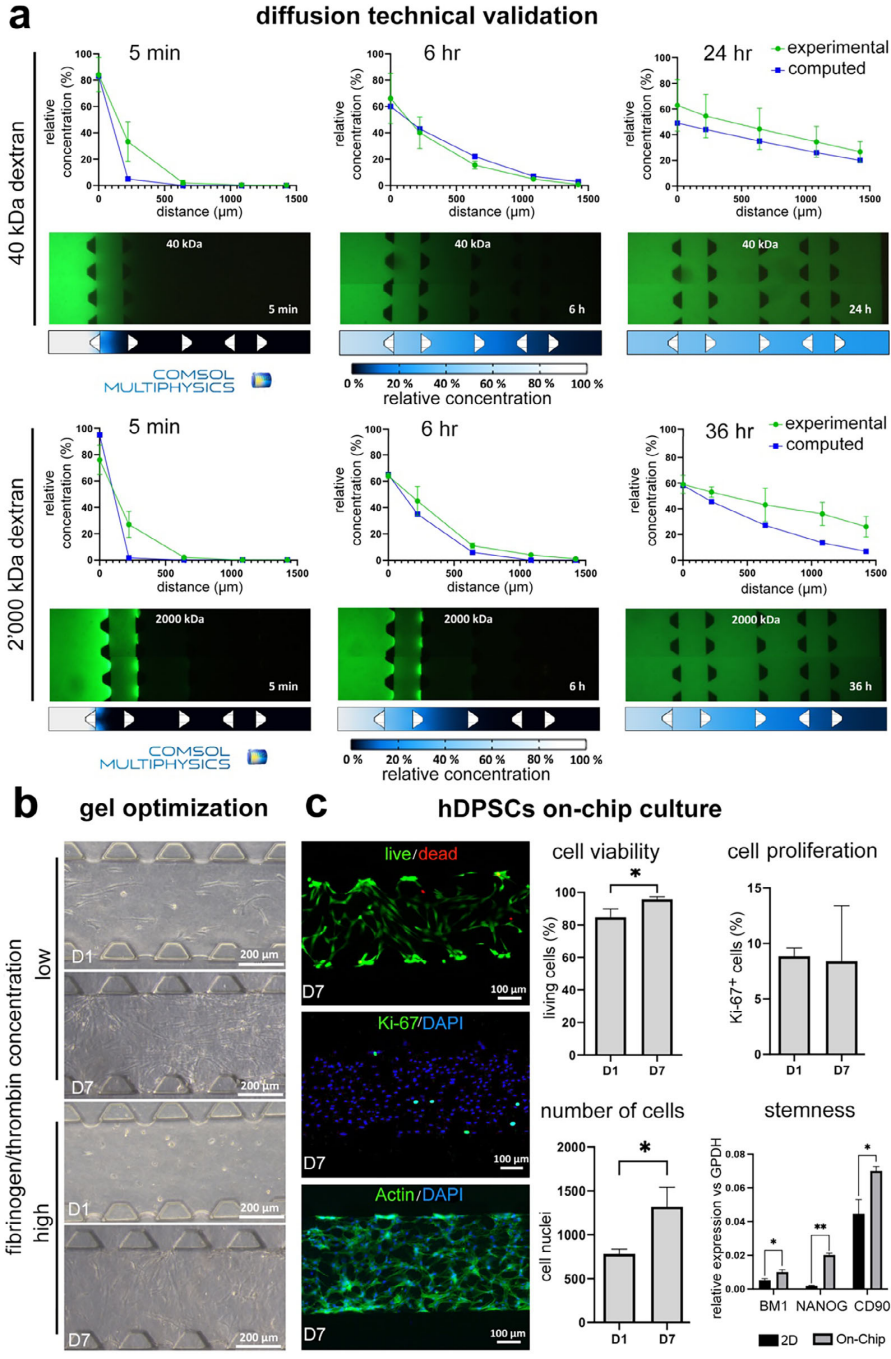
Technical validation and biological optimization. a) Dextran diffusion analysis. Percentage of the relative concentration of 40 and 2′000 kDa dextran in the device at 5 min, 6 and 24 or 36 h by experimental diffusion (green line) or computational procedure (blue line). Dextran concentration in the reservoirs was considered a 100% concentration value. Microscopy images and images of the computational results are shown. b) Fibrin gel concentration optimisation. Representative bright field image of hDPSCs embedded in fibrin gel on the chip. Two different hydrogels prepared by using different fibrinogen‐thrombin concentrations were tested (see Materials and Methods section). High‐concentration fibrin was chosen as optimal due to the control of cell proliferation and confluency. c) hDPSCs niche characterisation. Left, upper image: representative images of live (green) and dead (red) cells on day 7. Middle image: representative image of Ki‐67 expression (green) at day 7 (nuclei stained with DAPI, blue); lower image: representative fluorescence image of hDPSCs at day 7 of on‐chip culture (phalloidin in green marks F‐actin, while DAPI, in blue, marks cell nuclei). Right: quantification of the percentage of viable cells (10 x magnification; N = 3), percentage of Ki‐67 positive cells and the number of nuclei both over the culture period (N = 3), and stemness marker evaluation by *CD90, BMI1*, and *NANOG* expression by hDPSCs cultured on the chip for 7 days for 2D culture (data were normalized against the expression of the housekeeping GAPDH gene and expressed as average ± standard deviation; level of significance *p<0.05, **p<0.01).

Diffusion of metabolites and soluble factors is fundamental to ensure cell‐cell communication within “organ‐on‐chip” devices. Such molecules can diffuse differently within the various compartments and hydrogels included in the device, depending on the geometrical features of each chamber. A 3D computational model was developed to evaluate the mass transport, and in silico data were compared with diffusion experiments conducted with either 40 kDa or 2′000 kDa dextran over 24 or 36 h, respectively (**Figure** **2**a). The computational model predicted a slower diffusion of 40 kDa and 2′000 kDa dextran, 5.0% and 1.7%, respectively, in the first chamber after 5 min in comparison with experimental evidence (33.4 ± 15.0% and 27.0 ± 10.2%, respectively). Low molecular weight dextran showed a comparable diffusion kinetic reaching the microgrooves (distance = 1400 µm) in 6 h in both in silico analyses and experimentally (7.1% and 4.9 ± 1.0%, respectively). As expected, high molecular weight dextran required more time to cover the same distance (36 h), but it showed a faster diffusion (26.3 ± 8.0%) than computationally predicted (6.9%), particularly at later time points. This discrepancy can be caused by the diffusion coefficient used to perform the computational analysis and in the presence of slow convection fluxes that naturally occur throughout the gel. Furthermore, no dextran was measured in the area beyond the microgrooves (i.e., trigeminal ganglion compartment) at the last time point, further supporting the effectiveness of microgroove compartmentalisation. Overall, these results indicate that the geometry of our newly designed “tooth‐on‐chip” device can guarantee good mass transport.

### 2.2 Human Dental Pulp Stem Cells (hDPSCs) Preserve their Stemness and Proliferative Ability on the “Tooth‐On‐Chip” Device

We first optimized the composition of the fibrin hydrogel. 20 mg mL^−1^ of fibrinogen and 5 U mL^−1^ of thrombin were selected as optimal in terms of hDPSCs behavior, as this gel composition was able to control cell proliferation and extend the culture period to a minimum of 7 days before reaching confluency (Figure 2b, “high concentration”). Cells appeared uniformly distributed along the chamber, started to elongate one day after the seeding, and reached confluence after 7 days of culture. hDPSCs colonized the entire microfluidic chamber and exhibited the typical fibroblastic‐like shape, as confirmed by F‐actin immunostaining (Figure 2c). Viability and proliferation of hDPSCs in microfluidic devices were evaluated by live/dead assay and immunostaining against the Ki‐67 protein, respectively, on days 1 and 7 of culture. The analysis showed that hDPSCs were highly viable, with rates of 85.0 ± 5.2% and 96.0 ± 1.8% on both days 1 and 7, respectively (Figure 2C), while the cell proliferation profile was similar at these two‐time points (Figure 2c). The total cell counting showed that hDPSCs almost doubled after 7 days of culture in the “tooth‐on‐chip” device (Figure 2c). Moreover, the expression of *BMI1, NANOG*, and *CD90* in hDPSCs cultured for 7 days was higher, with fold change increases of 2.08, 11.04, and 1.76, respectively, when compared to standard 2D cultures of hDPSCs (Figure 2c).

### 2.3 Human Umbilical Vein Endothelial Cells (HUVECs) and hDPSCs Create 3D Vascularized Dental Pulp Tissue in the “Tooth‐On‐Chip” Device

**Figure 3:**
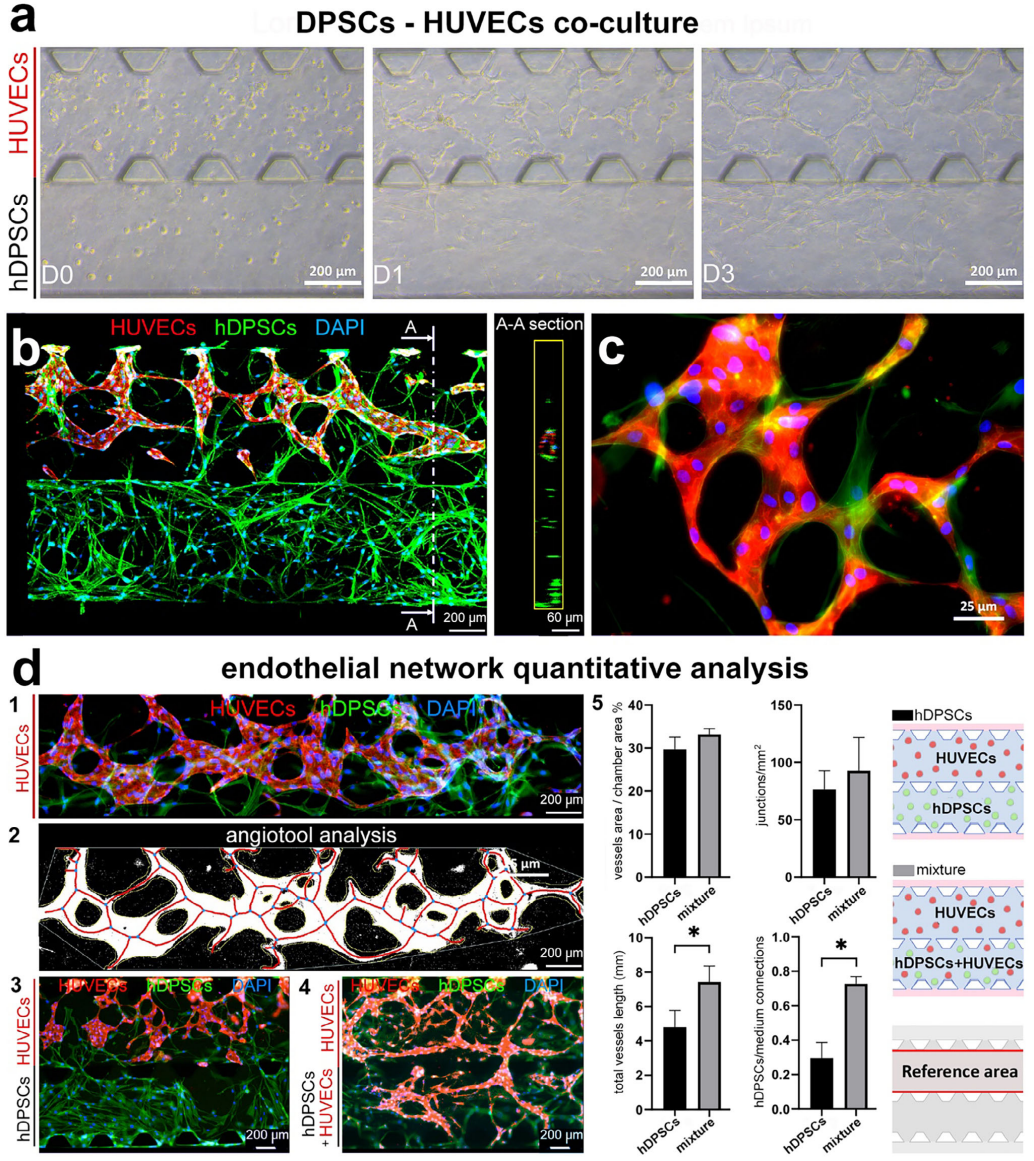
Vascularized dental pulp compartment. a) Representative bright field images of HUVECs and hDPSCs channels at days 0 (D0), 1 (D1), and 3 (D3). b) Representative image captured both at the front and at lateral (A‐A section) views of stained HUVECs and hDPSCs at day 4 with CD31 (red) and phalloidin (green) for F‐actin, respectively. Nuclei are visualized with DAPI (blue). c) A blow‐up of the formed vascular network showing connections (yellow) between hDPSCs (green) and HUVECs (CD31‐positive, red). Nuclei are visualized with DAPI (blue). d) Angio‐tool analysis on endothelial networks. d_1_: representative analysed picture, original IF image. hDPSCs in green, HUVECs in red, and cell nuclei in blue. d_2_: binary converted image with branches (red line), and junctions (blue dots). d_3_: vascular network within chambers of separately cultured hDPSCs (green) and HUVEC (red). Cell nuclei are in blue. d_4_: vascular network formation chambers containing mixed hDPSCs (green) and HUVEC (red), and HUVECs alone. Cell nuclei are in blue. d_5_: comparison of HUVECs‐hDPSCs separately seeded in adjacent channels and the mixed configuration. Endothelial network area, junctions, total branch length, and connections with lateral channels are shown. Level of significance *p<0.05.

To reproduce a vascularized dental pulp tissue compartment, HUVECs and hDPSCs were seeded in the chip in separate chambers. From day one, HUVECs elongated and self‐assembled into a vessel network whose branch numbers and sizes increased by day 3 (**Figure** **3**a), forming a well‐organised 3D vascular network within 4 days of culture. This network was composed of vessel‐like structures with empty lumens, as shown by CD31/F‐actin immunofluorescent co‐staining (Figure 3b; Figure S1, Supporting Information). At the same time, hDPSCs quickly proliferated and colonized the entire chamber but also migrated into the vascularized compartment (HUVECs) creating interconnections with the endothelial cells (Figure 3c). These migrating hDPSCs supported the growth of the endothelial cells and the formation of a vascular network (Figure 3b,c).

To further increase the vascularisation of the hDPSCs‐derived dental pulp tissue, HUVECs and hDPSCs were added to the same gel. Image analysis with the Angio‐Tool software was used to assess the behavior of HUVECs in terms of their migration and vascularisation capabilities when these cells were cultured alone or in the presence of hDPSCs (Figure 3d). When hDPSCs and HUVECs were co‐cultured in the same chamber, the length of the vascular network and the number of connections between hDPSCs and HUVECs significantly increased. No significant differences were found in the vascular network area and number of junctions per millimetre (Figure 3d). Based on these results, HUVECs and hDPSCs were co‐cultured in the same channel in our definitive “tooth‐on‐chip” system.

### 2.4 Dentine Induces hDPSCs Polarization and Enhances their Odontogenic Fate

**Figure 4:**
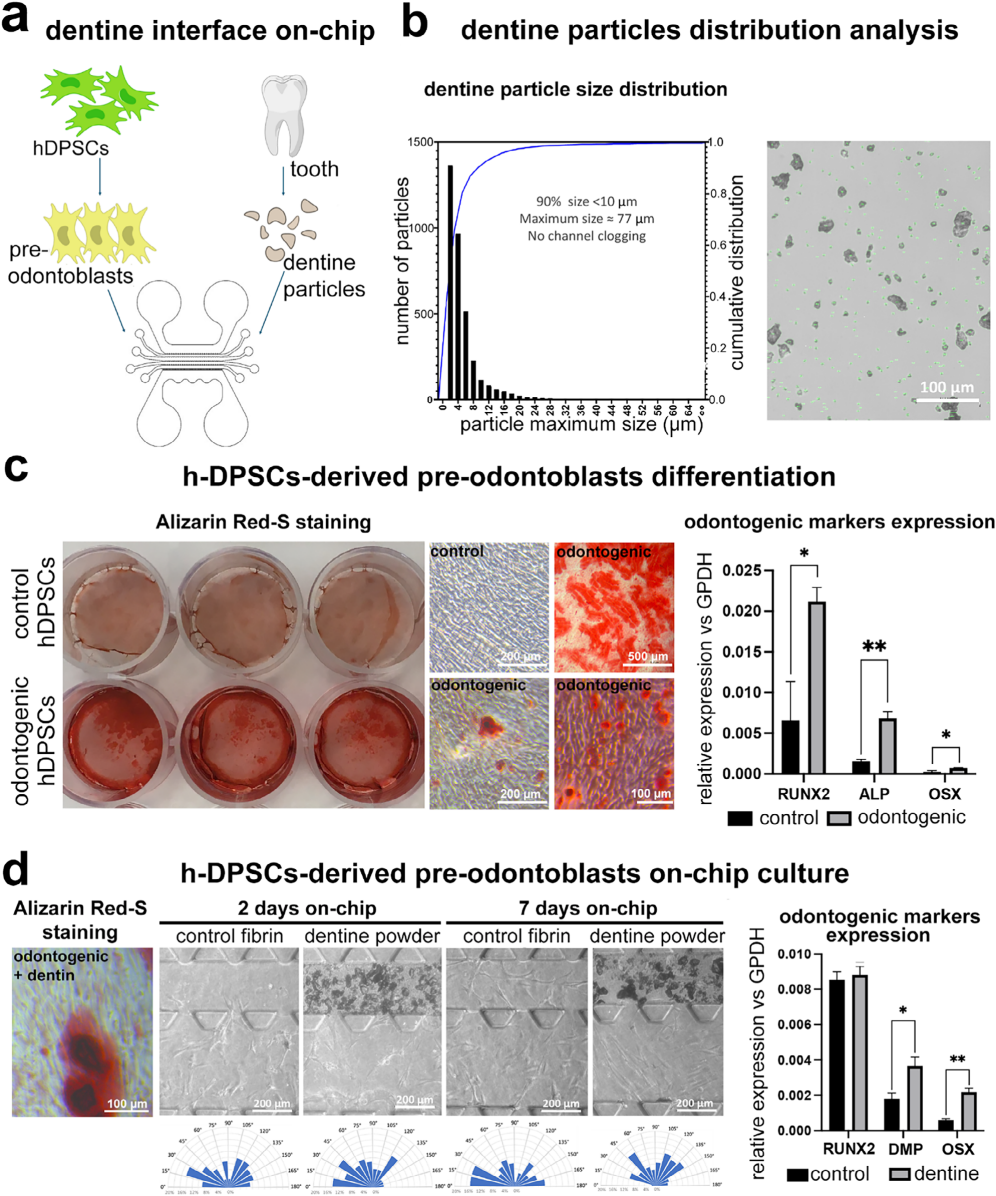
Dentine‐preodontoblasts interface. a) Schematic representation of the experimental approach. b) Left: dentine particle size distribution histogram. Size bins (2 µm range) are shown on the x‐axis, while the y‐axis shows the absolute frequency. The blue line shows the cumulative distribution curve. 90% of dentine particle size is lower than 10 µm, while the measured maximum size is 77 µm, therefore allowing for their loading in the microfluidic channel. Right: brightfield images of dentine powder suspension and particle analysis mask used for imageJ analyses. c) hDPSCs differentiation toward odontoblast‐like cells. Left: images of control and odontogenic hDPSCs stained with ARS. Right: gene expression of *RUNX2, ALP*, and *OSX* of hDPSCs treated with odontogenic medium for 30 days. Absolute expression was normalized against GAPDH and showed an average fold increase ± standard deviation compared with the control. d) Cell polarisation in the dentine‐odontoblast interface. Representative bright field images of odontoblastic cells cultured in the “tooth‐on‐chip” with fibrin gel only (control) or in the adjacent channel at 2 and 7 days. The orientation measured from od cultured in both dentine substitute and control chips at 2 and 7 days is shown in the radar charts. Right: expression of the odontogenic genes *RUNX2*, *DMP1*, and *OSX* in dentine substitute and control hDPSCs‐derived odontoblasts after 7 days of culture. Data were normalized against the expression of the housekeeping GAPDH gene and expressed as average value ± standard deviation. Level of significance *p<0.05, **p<0.01.

The dentine‐odontoblast interface was modeled by culturing hDPSCs‐derived pre‐odontoblasts in contact with dentine‐derived fragments (dentine powder) in our “tooth‐on‐chip” microfluidic device (**Figure** **4**a). The dentine powder, composed of particle sizes ranging from 3–77 µm, was suspended in the fibrin‐based hydrogel, thus creating a composite material compatible with microfluidic channels (Figure 4b). hDPSCs‐derived pre‐odontoblasts (Figure 4c) were also embedded into the hydrogel and plated in the adjacent dentine powder channel of the microfluidic system. “Tooth‐on‐chip” devices composed of hDPSCs‐derived pre‐odontoblasts and fibrin gel, but without the dentine powder component, were used as controls. After two days of culture, hDPSCs‐derived pre‐odontoblasts started to elongate, exhibited a fusiform shape, and were distributed with an isotropic orientation with no significant morphological differences between the two culture setups (Figure 4d). However, after 7 days of culture, only hDPSCs‐derived pre‐odontoblasts co‐cultured with dentine powder were preferentially orientated toward the mineralized compartment. Contrarily, cells cultured alone (without dentine powder) were randomly distributed, and some migrated toward the adjacent fibrin channel. We then assessed the expression of genes involved in odontoblast differentiation in hDPSCs‐derived pre‐odontoblasts after 7 days of culture in the presence or absence of dentine powder. Our data showed significantly higher levels of *DMP1* and *OSX* expression in cells cultured in the presence of dentine powder compared to cells cultured only in fibrin. The levels of *RUNX2* expression were like those observed in the cells cultured only in fibrin (Figure 4d).

### 2.5 Trigeminal Innervation of the 3D Vascularized Dental Pulp Tissue in the “Tooth‐On‐Chip” Device

**Figure 5:**
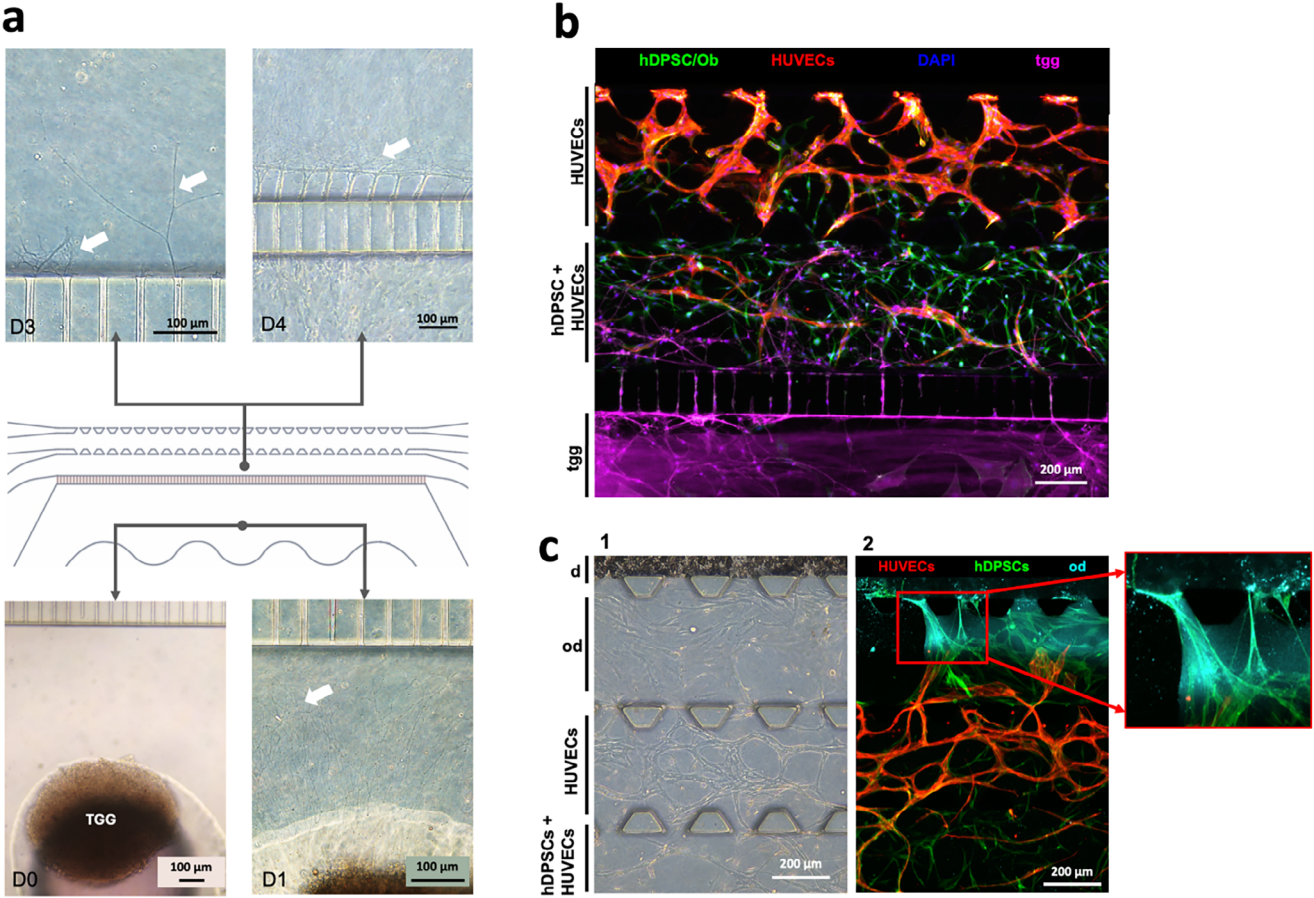
Completed “tooth‐on‐chip” device. a) Trigeminal ganglion (tgg) culture in the microfluidic device. Representative bright field pictures of the tgg on the day of the isolation (day 0; D0) and D1, and of axonal outgrowth into the microgrooves at D3 and D4. White arrows show growing axons. b) Immunofluorescent staining showing the innervated and vascularized dental pulp compartments within the microfluidic device: HUVECs (CD31‐positive cells in red), hDPSCs (visualized by F‐actin expression with phalloidin in green), and tgg (tdTomato fluorescent protein in purple), all counterstained with DAPI (blue). c) Representative bright field (c_1_) and immunofluorescence (c_2_) pictures showing the dentine‐odontoblast‐like cells interface and the dental pulp tissue compartment within the microfluidic device: HUVECs (CD31‐positive cells in red), hDPSCs (visualized by F‐actin expression with phalloidin in green), functional hDPSCs‐derived odontoblast‐like cells (od) (DSPP‐positive cells in cyan), and dentine (d) (stained by DSPP in cyan).

In the final and complete version of the “tooth‐on‐chip”, we integrated trigeminal ganglia, responsible for the innervation of the dental pulp in vivo and closely associated with both perivascular niches and odontoblasts.^[^ ^74^ ^]^ Trigeminal ganglia from mouse embryos were isolated and cultured in the neuronal compartment of the microfluidic device. The ganglia rapidly adhered to the bottom of the chamber, and axonal extension was visible after one day of seeding (**Figure** **5**a). On day 4, the microfluidic system was seeded with HUVECs, hDPSCs, hDPSCs‐derived pre‐odontoblasts, and dentine powder. At the same time, axonal projections from the trigeminal ganglion crossed the microgrooves, invaded the compartment where HUVECs and hDPSCs were seeded, and started to be part of the vascularized stem cell niches (Figure 5b; Video S1, Supporting Information). In addition, some HUVECs migrated toward the hDPSCs‐derived pre‐odontoblasts channel and established cell‐cell connections (Figure 5c; Video S2, Supporting Information). The hDPSCs‐derived pre‐odontoblasts did not exhibit the characteristic mesenchymal stem cell morphology and showed a preferential vertical orientation toward the dentine powder. Dentine fragments, visible by DSPP staining, started to leach through the gel and invaded the hDPSCs‐derived pre‐odontoblastic compartment.

## 3 Discussion

In this study, we tackled the challenge of creating a state‐of‐the‐art dental pulp emulation system by designing a “tooth‐on‐chip” device that allows co‐culture and functional communication between dental pulp stem cells, vasculature, innervation, and a dentine‐emulating compartment. To mimic the extracellular matrix of the dental pulp tissue, we used a fibrin hydrogel, a solution that provides a suitable in vitro environment for hDPSCs physiology, support vessel network self‐assembly, and can be easily remodeled and substituted by self‐produced extracellular matrix.^[^ ^75^, ^76^, ^77^, ^78^, ^79^ ^]^ Indeed, hDPSCs showed a typical fibroblastic‐like morphology, high cell viability, and a good proliferation rate. Compared to standard 2D cultures, hDPSCs cultured in fibrin hydrogel in our microfluidic system expressed higher levels of *CD90*, *BMI1*, and *NANOG*, genes important for stem cell multipotency and self‐renewal.^[^ ^9^, ^80^, ^81^ ^]^ The co‐culture of hDPSCs and endothelial cells (HUVECs) in the “tooth‐on‐chip” device allowed the generation of capillary structures that form a dense vascular network within its dental pulp chamber. The vascular network exhibited hollow lumens, while migration of hDPSCs toward the endothelial chamber allowed their interaction with the already‐formed vascular structures. The vasculature was not homogeneously distributed along the microfluidic chamber, exhibiting a high variability in the length and lumen diameter of the vessels. Stable and intensive angiogenic sprouting was co‐localized in areas populated by hDPSCs that act as supportive cells. The contact between hDPSCs and endothelial cells closely resembles and emulates the perivascular dental pulp stem cell niches in vivo.^[^ ^4^, ^5^, ^82^, ^83^, ^84^ ^]^ The present results agree with previous findings on co‐cultured endothelial cells with either pericytes or hPDLSCs in microfluidic devices.^[^ ^69^, ^85^ ^]^

Implementation of the mineralized compartment of our novel “tooth‐on‐chip” system was based on two adjacent channels: one containing pre‐odontoblasts already differentiated from hDPSCs, and the other filled with dentine‐derived material. Differentiated hDPSCs‐derived odontoblasts were polarised and orientated in the same direction toward the dentine chamber, mirroring the morphological appearance of odontoblasts and the spatial organisation of the odontoblastic layer of healthy human teeth.^[^ ^86^ ^]^ This is significant since emulating the in vivo structure of the odontoblastic layer is crucial to promoting human dentine formation and dental tissue regeneration.^[^ ^87^ ^]^ Furthermore, previous studies using either decellularized dentine surfaces or dentine‐composite materials have highlighted the importance of dentine in generating hDPSCs‐derived odontoblast‐like cells.^[^ ^88^, ^89^ ^]^ This was further supported by the upregulation of genes involved in odontoblast differentiation, such as *RUNX*2, *DMP1*, and *OSX*,^[^ ^90^, ^91^ ^]^ when hDPSCs‐derived odontoblast‐like cells were co‐cultured with dentine. The dentine chamber implementation in our “tooth‐on‐chip” system poses the grounds for modeling dental pathologies such as early stages of carious decay progression within the pulp, where the odontoblast‐dentine interface plays a pivotal role.

The last key addition to our microfluidic system was trigeminal innervation. Trigeminal innervation conveys tooth pain and sensation and is actively involved in dental pulp tissue regeneration upon tooth injury.^[^ ^92^, ^93^ ^]^ Trigeminal ganglion axons rapidly sprouted, reached the dental pulp chamber of the microfluidic system, established contacts with hDPSCs and the HUVECs‐derived vascular network, and formed a stable 3D neuronal plexus. These results agree with our previous findings, showing the axonal growth and network formation of trigeminal neurones co‐cultured with hDPSCs in microfluidic devices.^[^ ^54^, ^55^ ^]^ The incorporation of innervation in our “tooth‐on‐chip” system poses the basis for its utilisation to investigate the crosstalk between neurones and all other dental pulp components. We chose to include mouse trigeminal ganglia as to date, and despite minor species‐specific differences,^[^ ^94^ ^]^ they represent the most physiologically relevant model of human trigeminal ganglia available. Protocols to generate trigeminal neurons for human induced pluripotent stem cells (hiPSCs) are available.^[^ ^95^ ^]^ While promising, these hiPSCs‐derived trigeminal neurons have limitations, as they have immature electrophysiological profiles and altered sensitivity to stimuli, which drastically reduce their physiological relevance.^[^ ^96^ ^]^ Furthermore, such neurons have so far only been studied as neuronal 2D cultures, lacking the 3D microenvironment and extracellular matrix (ECM) components that are central for proper function. In vivo, the trigeminal ganglion consists of multiple subtypes of sensory neurons (nociceptors, mechanoreceptors, proprioceptors), and satellite glial cells, distributed in precise 3D topographies in a defined ECM microenvironment,^[^ ^94^ ^]^ all features that would lack in a 2D culture of hiPSCs‐derived trigeminal neurons. Future efforts will be dedicated to generating a complex model of a human trigeminal ganglion that would provide a completely human‐derived alternative to mouse‐derived tissues.

Our microfluidic device design makes it suitable for investigating different aspects of dental pulp pathophysiology. From the beginning of the culture, it represents a good model to assess regenerative processes within the dental pulp. The generation of the device indeed relies on the co‐culture of several separate elements that undergo a complex set of self‐organization to recreate in vivo‐like tissue structures. The trigeminal ganglia used in this device have undergone axonal damage during dissection and innervate the dental pulp compartment of the chip via projection of their regenerating axons. Dental pulp stem cells, isolated originally from adult human teeth, interact with co‐cultured endothelial cells to re‐organize themselves into perivascular niches, and trigeminal neurons innervate their targets in this highly dynamic environment reminiscent of regenerative processes. The model is considered as settled seven days after the start of the culture. At this time point, the structure of the pulp tissue was well‐organized, with an endothelial network supported by hDPSCs, which similarly to the in vivo condition, act as pericytes along the formed vessels.^[^ ^3^, ^82^ ^]^ Furthermore, axonal networks were formed, with trigeminal neurons innervating both the pulp and the odontoblastic compartment, thus closely resembling the in vivo situation.^[^ ^97^ ^]^ This timepoint can thus be considered appropriate for testing drugs, materials, and other therapeutic molecules in a dental pulp tissue. To this end, the characterization of molecular diffusion on our device provides an important body of information to design proper screens of drugs and materials, a key application of “organ‐on‐chip” devices.^[^ ^38^ ^]^ The present microfluidic device further provides a promising platform for the implementation of pathological conditions, ranging from bacterial infections to traumatic injuries, to study reparative and regenerative processes.

Under normal physiological conditions, the dental pulp is a relatively quiescent tissue. Blood vessels supply essential nutrients, while dental pulp stem cells are predominantly found in perivascular niches in a dormant state until activated by injury^[^ ^3^, ^98^ ^]^ Fibroblasts and other mesenchymal cells participate minimally in extracellular matrix remodeling, odontoblasts secrete small amounts of secondary dentine, and nerve fibers innervate the tissue without producing consciously perceptible sensory outputs.^[^ ^99^ ^]^ This steady state is replicated in the present microfluidic system, since in the dental pulp compartment the DPSCs are organized into 3D perivascular niches adjacent to the vessel‐forming endothelial cells that are fully innervated by trigeminal ganglia in a realistic topography. To date, the present microfluidic model represents the most advanced dental pulp emulation system published and the first to integrate key components such as DPSCs, trigeminal innervation, blood vessels, perivascular niches, pre‐odontoblasts, and dentine.^[^ ^100^ ^]^ Its unique design recapitulates the basic functional interactions that characterize the healthy dental pulp tissue. Other functions, such as observation of neuronal activity on the chip device, will require the establishment of injury and disease models, and will be the basis for future lines of research. Other processes, such as caries progression and secondary dentine matrix deposition, occur at too slow paces that are not observable on the temporal scale of our experiments. Indeed, the establishment of a long‐term “organ‐on‐chip” dental pulp emulation device would be of great interest for the investigation of pathophysiological processes occurring on longer time scale, such as dental pulp ageing and slow caries progression events. Long‐term culture is in general a key challenge for most “organ‐on‐chip” devices^[^ ^38^ ^]^ and will require the integration of several new components. First, the use of a perfusable microfluidic device, i.e., the integration of a system for continuous medium circulation, either as an external apparatus or integrated within the chip. Second, and most challenging, would be the creation of a cytostatic environment for the 3D vascularized dental pulp, i.e., a durable, non‐degradable extracellular matrix that would support the survival of hDPSCs and endothelial cells for a long period of time in a dormant‐like state that would prevent the overfilling of the culture channel. Another potential point of improvement is the inclusion of actual dentine, or a mineralized dentine‐emulating compartment. Dentine powder, used in our study, is known to release dentine‐matrix derived factors that can promote, even in the absence of an actual dentine structure, odontoblasts differentiation and stabilization.^[^ ^101^, ^102^ ^]^ Dentine powder is compatible with a completely miniaturized and injectable chip design, such as the one we proposed in this study, a feature that makes this design highly scalable and automatable. In line with previous knowledge, we indeed observed that odontoblasts cultured in the presence of dentine powder expressed higher levels of odontoblastic differentiation markers and showed more pronounced polarization. On the other hand, dentine powder does not recapitulate the mechanical and structural properties of dentine, and it is a suboptimal solution when assessing the response of dental tissues to materials, or emulating host‐microbiome interactions, all scenarios that will require inclusion of dentine, or a compartment emulating all the features of actual dentine.

These and other exciting improvements could be implemented in future studies on the currently presented design, which at now stands as the most advanced platform for the emulation of healthy dental pulp tissue.

In conclusion, we created a multifaceted microfluidic model system of the human dental pulp, which includes hDPSCs, polarised odontoblast‐like cells, dentine, vascular cells, and neurones. The present “tooth‐on‐chip” device represents a powerful tool for modeling the structural complexity of the dental pulp tissue and emulating its physiopathology. This innovative platform can be already used for studies focused on diverse topics, ranging from dental tissue regeneration strategies to drug screening and biomaterial testing.

## 4 Experimental Section

### Device Design and Computational Model

**Figure 6:**
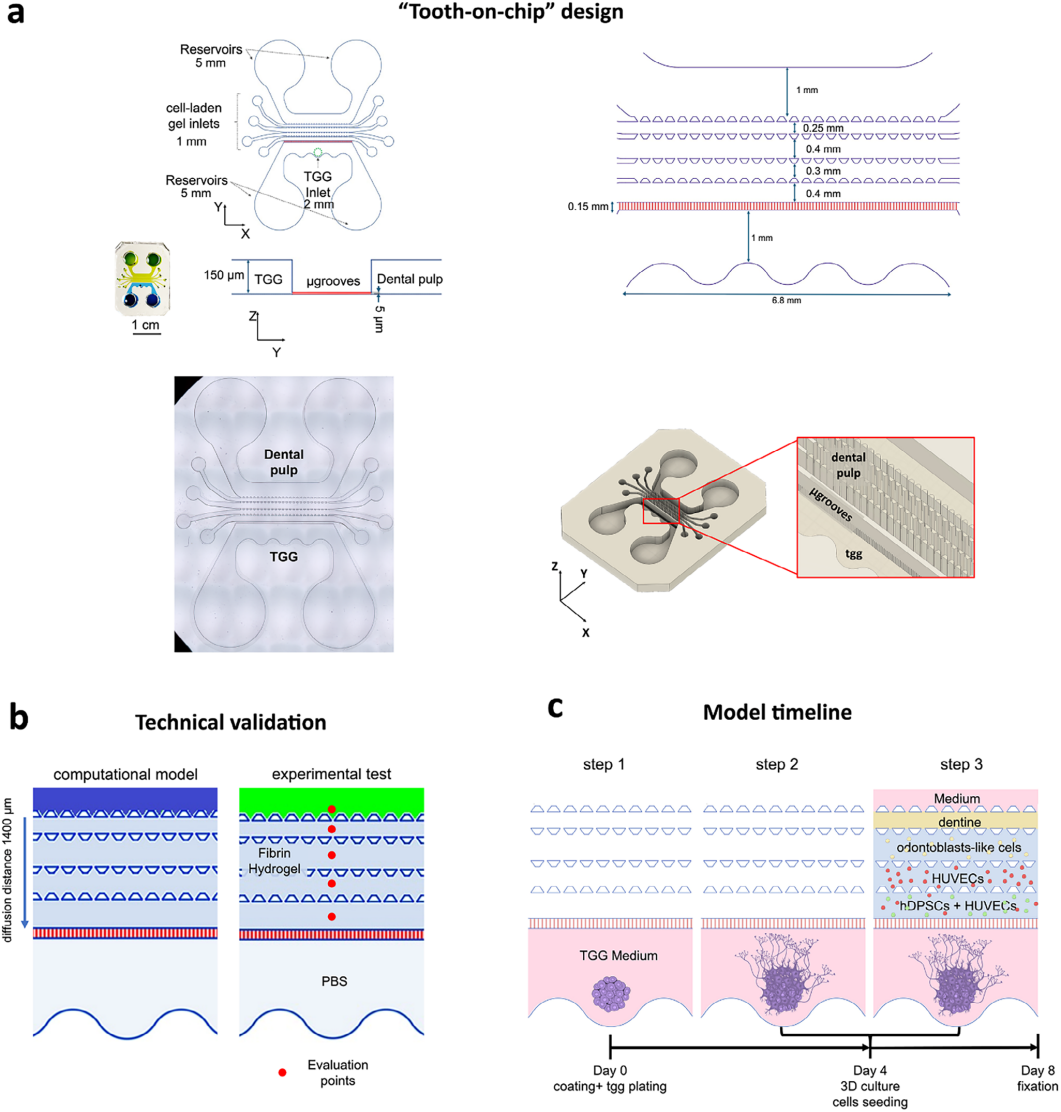
Tooth‐on‐chip design, technical and biological validation. a) Top: tooth‐on‐chip design. Illustrations of the top and side views of the model indicate the position and dimension (µm) of the dental pulp and trigeminal ganglion (tgg) compartments in the chip. Bottom: Brightfield image of the whole device and 3D representation of the chip showing the µgrooves connecting the two compartments. b) Schematic representation of the computational model for diffusion studies and dextran diffusion experimental set‐up. c) Schematic representation of the chip seeding procedure. On the first (day 0), the tgg chamber was coated with poly‐D‐lysine and laminin, and then the isolated trigeminal ganglia were seeded. The axons grew, and when they reached the microgrooves (days 3–4), hDPSCs, HUVECs, odontoblasts, and dentine were seeded on the device and cultured for four additional days.

The “tooth‐on‐chip” microfluidic device layout was drawn using computer‐aided design (CAD) software (AutoCAD, AutoDesk Inc.). The device features two primary compartments (**Figure** **6**a): the neuronal compartment (“tgg compartment”) for isolated trigeminal ganglia (tgg) culture, and the dental pulp compartment, which houses a 3D culture of cell‐laden hydrogels. Both compartments are 150 µm high and are linked by microchannels (microgrooves, highlighted in red) designed to prevent the migration of whole‐neuronal cells into the dental pulp region. These microgrooves are 150 × 10 × 5 µm (length x width x height) and are separated by a 50 µm gap. The tgg compartment is a single chamber with a 2 mm inlet for tgg plating and two 5 mm reservoirs for medium supply. The pulp compartment is divided into five channels separated by four rows of trapezoidal pillars. These channels sequentially contain: culture medium, dentine particles, human dental pulp stem cells (hDPSCs), human umbilical vein endothelial cells (HUVECs), and hDPSCs‐derived odontoblast‐like cells (od) (Figure 6a). Each channel has two 1 mm ports for gel injection, except for the medium channel, which has 5 mm reservoirs at both ends.

The dimensions of the microfeatures were designed upon the analysis of diffusion phenomena: the microfluidic device design was converted into a 3D computational model to assess the ability of specific molecules to diffuse within the different channels of the pulp compartment. A defined spatial domain (1400 µm) spanning from the medium channel to the microgrooves was considered for the analysis (Figure 6b). Diffusion dynamics of small molecules (e.g., nutrients, growth factors) and larger species (e.g., exosomes and liposomes) was studied by varying the molecular weight parameter used for the simulation. 40 kDa dextran (for modeling small molecules) and 2′000 kDa dextran (for mimicking large compounds) were considered for the simulation, so to also have the possibility to confirm the computational data with experimental results. Simple diffusion without convection was chosen as transport mechanisms to consider the least efficient condition for mass transport. Furthermore, no dextran adsorption on PDMS and neglectable diffusion phenomena at the zone near the reservoirs were considered to simplify the model. Diffusion coefficients for different molecular weights were found in literature.^[^ ^103^, ^104^ ^]^ Computations were performed using COMSOL Multiphysics (COMSOL Inc.).

The “tooth‐on‐chip” device was made of polydimethylsiloxane (PDMS; Sylgard 184; Dow Corning, 01064291z) and included both a top microfluidic layer patterned with the micro‐sized features and a flat bottom layer. Multiple copies of the device were fabricated through soft‐lithography techniques.^[^ ^105^ ^]^ Briefly, a silicon master mould was fabricated using soft lithography methods. The silicon master mould was produced in a clean room environment (PoliFAB, Politecnico di Milano) using maskless photolithography techniques. A 5 µm‐thick layer of negative photoresist (SU‐8 2005; Microchem, USA) was spin‐coated onto a 6′’ wafer, and microgrooves features were cross‐linked by direct laser writing (Heidelberg MLA100, Heidelberg Instruments). Then two 75 µm‐thick layers of negative photoresist (SU‐8 2050; Microchem, USA) were spin‐coated and cross‐linked one after the other to achieve the total 150 µm chamber layer height (tgg and dental pulp compartments). Once the exposure was completed, the non‐cross‐linked photoresist was removed with a developer solution (SU‐8 developer; Microchem). The resulting wafer had the negative features of the desired microfluidic PDMS device. The “tooth‐on‐chip” was produced by pouring PMDS on the master mould and curing it at 65 ^°^C for 3 h. The PDMS layer was peeled off and bonded via air plasma treatment onto a flat PDMS membrane. Light microscopy (Leica DM6000 B microscope) was used to measure the microscale features and assess the quality of the adopted production protocol.

### Dextran Diffusion Tests

Computational results were compared to experimental tests performed with fluorescent dextran according to the following setup (Figure 6b): each pulp compartment channel was separately filled with fibrin‐based hydrogel (i.e., Fibrinogen 20 mg mL^−1^, Thrombin 5 U mL^−1^ in PBS; Sigma‐Aldrich/Merck, Germany), and the device was incubated for 5 min at 37 ^°^C for gelation. The upper medium channel was filled either with 40 kDa FITC‐Dextran solution (i.e., 0.5 mg mL^−1^; Sigma‐Aldrich/Merck, FD40) or with 2000 kDa FITC‐Dextran solution (i.e., 0.5 mg mL^−1^; Sigma‐Aldrich/Merck, FD2000S), whilst in the tgg compartment PBS (phosphate buffered saline, ThermoFisher) was added.

Samples were incubated at 37 ^°^C and images were taken every 30 min using a Leica DM6000 microscope equipped with a Leica DFC350FX camera and the Leica Application Suite Advanced Fluorescence (LAS AF) software. Images were analysed with Fiji software^[^ ^106^ ^]^ and fluorescence intensity was measured across the chambers. Fluorescence‐concentration linear dependence and reservoir intensity were used to compute and normalize the relative concentration in the chambers.

### Isolation and Culture of Human Dental Pulp Stem Cells (hDPSCs)

The procedure for anonymised human teeth collection at the Centre of Dental Medicine, Zurich, was approved by the Kantonale Ethikkommission of Zurich, and the patients gave their written informed consent (reference number 2012‐0588). All procedures were performed according to the current guidelines. hDPSCs were isolated from dental pulps of extracted teeth of healthy patients. Dental pulps were enzymatically digested in a solution composed of collagenase A (3 mg mL^−1^; Life Technologies Europe BV, 50‐100‐3278) and dispase (4 mg mL^−1^; Sigma‐Aldrich/Merck, D4818) for 1 h at 37 ^°^C with gentle agitation. The digested tissue was filtered with a 70 µm Falcon filter. After centrifugation, the cell suspension was cultured in a growth medium consisting of Dulbecco's modified eagle medium/nutrient mixture F‐12 (DMEM/F12; ThermoFisher, 31330038) supplemented with 10% of foetal bovine serum (FBS), penicillin/streptomycin (pen/strep: 100 U mL^−1^–100 µg mL^−1^, ThermoFisher, 15140148). Cells were plated in T25 flasks at 37 ^°^C, 5% CO_2_, and the medium was changed twice a week. After reaching 80% confluency, cells were harvested using trypsin/EDTA (0.05% trypsin, 0.02% EDTA; ThermoFisher, 15400054) and plated for expansion. Isolated hDPSCs at passages IV‐VIII were used for experiments.

### HUVECs Culture

Commercially available HUVECs (Lonza, C2519A) were cultured in endothelial growth medium (EGM‐2) (bullet kit; Lonza CC‐3162) supplemented following the manufacturer's instructions. All experiments were performed using HUVECs between passages IV‐VI.

### Vascularized Dental Pulp Compartment

To generate a 3D vascularized compartment within the “tooth‐on‐chip”, human fibrinogen (Sigma‐Aldrich, F3879) and thrombin (Sigma‐Aldrich, T6884) were mixed with cell suspension according to the desired final concentration. To set up the optimal fibrin composition for hDPSCs culture, the hydrogel pre‐polymer solution was loaded with cells at a final concentration of 1∙10^6^ cells mL^−1^ and cultured in the microfluidic device using two different fibrinogen‐thrombin concentrations, namely low (10 mg mL^−1^–2.5U mL^−1^) and high (20 mg mL^−1^–5U mL^−1^). The culture was carried out for one week, and the medium was changed every day. To emulate vascularized dental pulp, hDPSCs and HUVECs were co‐cultured on the chip. To this end, the HUVECs chamber was loaded with a HUVECs‐laden hydrogel containing 6∙10^6^ cells mL^−1^ in 5 mg mL^−1^ fibrinogen – 2 U mL^−1^ thrombin. The adjacent channel was loaded with either hDPSCs alone (1∙10^6^ cells mL^−1^) or with hDPSCs (1∙10^6^ cells mL^−1^) and HUVECs (3∙10^6^ cells mL^−1^) in 20 mg mL^−1^ fibrinogen – 5 U mL^−1^ of thrombin. The co‐culture was maintained in a medium composed of a 1:1 mixture of hDPSCs medium and HUVECs medium. Cells were cultured for four days with daily medium replacement.

### Dentine‐Odontoblasts Interface Compartment

Extracted human premolars were broken into two parts and cleaned from soft tissue residuals to obtain the dentine powder. The enamel was removed by a diamond saw from the upper part of the crown and discarded. The collected hard tissue was stored in 70% ethanol at 4 °C until use. Eventual immunogenic material was removed by incubation in NaClO 0.5% for 24 h at room temperature (RT). After being thoroughly washed in deionized water, samples were dipped in 70% ethanol and dried. Finally, the dentine powder was obtained by smashing the tooth halves in a metal mortar‐pestle system filled with liquid nitrogen. The finest powder was obtained after filtration through a 40 µm strainer and then UV‐sterilized. The prepared powder was resuspended in PBS at the final concentration of 150 mg mL^−1^ and supplemented with pen/strep and 2.5 µg mL^−1^ Amphotericin B (Sigma‐Aldrich, A2411). Particle size distribution in the powder suspension was analysed by imaging using light microscopy (Leica DM6000 microscope) and measured by ImageJ software. To model the dentine‐odontoblasts interface, hDPSCs were differentiated toward the odontoblastic lineage before their culture in the microfluidic device. Briefly, hDPSCs were plated in T75 flasks at a cell density of 10^4^ cells cm^−2^ in a growth medium. After 3 days of culture, hDPSCs reached confluence and were cultured for 30 days in odontogenic inductive medium, consisting of Dulbecco's Modified Eagle Medium/Nutrient Mixture F‐12 (DMEM/F12; ThermoFisher, 31330038) supplemented with 10% of foetal bovine serum (FBS), penicillin/streptomycin (pen/strep: 100 U mL^−1^–100 µg mL^−1^, ThermoFisher, 15140148), 10 nM dexamethasone (Sigma‐Aldrich, D2915), 10 mM β‐glycerophosphate (Sigma‐Aldrich, G9891), and 50 µg mL^−1^ ascorbic acid (Sigma‐Aldrich, A8960). The hDPSCs differentiation potential was assessed by Alizarin‐Red S staining (Sigma‐Aldrich, A5533) and by odontogenic gene expression analysis via real‐time PCR. Differentiated hDPSCs‐derived odontoblast‐like cells and dentine particles were embedded in the hydrogel (2 × 10^6^ cells mL^−1^ in 20 mg mL^−1^ fibrinogen – 5 U mL^−1^ thrombin) and separately injected into their specific designed channel. The same fibrin composition used for the hDPSCs channel was applied to the pre‐odontoblast channel. The mechanical properties of the dentine‐composite hydrogel were not considered to impact cell behavior, as the cells are not embedded in the same material. Dentine was solely utilized to release pre‐odontogenic factors. hDPSCs growth medium was added in the medium channels after 8 min of incubation. As a control, samples with fibrin gel‐only (20 mg mL^−1^ fibrinogen, 5 U mL^−1^ thrombin) were used instead of dentine powder in the dentine channel. hDPSCs‐derived odontoblast‐like cells were cultured on a chip with hDPSCs growth medium for 7 days, with media change occurring every other day. Dentine samples (n = 3) and control samples (n = 3) were imaged with bright field optical microscopy, and Fiji software^[^ ^106^ ^]^ was used to evaluate cellular polarisation and orientation.

### Animal Handling

All mice were maintained and handled according to the Swiss Animal Welfare Law and in compliance with the regulations of the Cantonal Veterinary Office, Zurich (ZH205/2020). The animal facility provided standardized housing conditions, with a mean room temperature of 21 ± 1 °C, 50% ± 5% humidity, and 15 complete changes of filtered air per hour (HEPA H 14filter); air pressure was controlled at 50 Pa. The light/dark cycle in the animal rooms was set to a 12‐h/12‐h cycle (lights on at 07:00, lights off at 19:00) with artificial light of ≈40 Lux in the cage. The animals had unrestricted access to sterilized drinking water and ad libitum access to a pelleted and extruded mouse diet in the food hopper (Kliba No. 3436; Provimi Kliba / Granovit AG, Kaiseraugst, Switzerland). Mice were housed in a barrier‐protected specific pathogen‐free unit and were kept in groups of max. Five adult mice per cage in standard IVC cages (Allentown Mouse 500; 194 mm × 181 mm × 398 mm, floor area 500 cm2; Allentown, New Jersey, USA) with autoclaved dust‐free poplar bedding (JRS GmbH + Co KG, Rosenberg, Germany). A standard cardboard house (Ketchum Manufacturing, Brockville, Canada) served as a shelter, and tissue papers were provided as nesting material. Additionally, crinklets (SAFE crinklets natural, JRS GmbH + Co KG, Rosenberg, Germany) were provided as enrichment and further nesting material. The specific pathogen‐free status of the animals was monitored frequently and confirmed according to FELASA guidelines by a sentinel program. The mice were free of all viral, bacterial, and parasitic pathogens listed in FELASA recommendations.^[^ ^107^, ^108^ ^]^ R26*^mT/mG^* (MGI: 3716464) mice (The Jackson Laboratory) expressing tdTomato fluorescent protein were time‐mated. Successful mating was assessed by vaginal plug check, and the day of the plug was considered the day of embryonic development 0.5 (E0.5). At E14.5, pregnant females were first anaesthetized by intraperitoneal injection with pentobarbital 200 mg kg^−1^. Depth of anaesthesia was assessed by the absence of reflexes, from tail to toe pinch reflex. After such assessment, anaesthetized pregnant females were euthanized via cervical dislocation followed by decapitation. Embryos were placed in cold PBS, and tgg were isolated as previously described.^[^ ^109^ ^]^

### Dental Pulp Innervated Compartment and Complete “Tooth‐On‐Chip”

The procedure of the four steps encompassing the seeding of tgg, HUVECs, hDPSCs, hDPSCs‐derived odontoblast‐like cells (od) and dentine substitute on‐chip is summarized in Figure 6c. First, the tgg compartment was coated with 0.2 mg mL^−1^ Poly‐D‐Lysine (ThermoFisher, A3890401) diluted in deionized water and 5 µg mL^−1^ laminin (Sigma‐Aldrich L6274) diluted in the neurobasal medium for 1 h at 37 ^°^C each. Then, the tgg medium, consisting of the neurobasal medium, B27 supplement (Gibco‐ThermoFisher), 2 mM L‐Glutamine (Gibco‐ThermoFisher, 17504044), 100 U mL^−1^ penicillin and 100 µg mL^−1^ streptomycin, 50 ng mL^−1^ nerve growth factor (R&D Systems, 256‐GF) and 0.25 pM cytosine arabinoside (AraC, Sigma‐Aldrich/Merck, C1768), was added. As a last step, tgg were isolated and immediately seeded (step 2) and cultured for four days with daily medium replacement after the second day (step 3). After that period, HUVECs, hDPSCs, dentine, and od‐laden hydrogels were added to the device in the respective channels (step 4) according to the optimized procedure established before. The final “tooth‐on‐chip” was cultured for four additional days with daily medium replacement consisting specifically of tgg medium for the tgg compartment and of hDPSCs‐HUVECs culture medium in a 1:1 ratio for the dental pulp compartment.

### Live/Dead Assay

To assess hDPSCs viability on the chip, cells were seeded in the hDPSCs chamber, and live/dead assay was performed after 1 and 7 days of culture. Samples were incubated for 10 min at room temperature (RT) with 8 ng mL^−1^ fluorescein diacetate (FDA; Sigma‐Aldrich/Merck, C‐7521) and 20 ng mL^−1^ propidium iodide (PI; Sigma‐Aldrich/Merck, P4170) in PBS. Images of live (green) and dead (red) cells were acquired with a Leica DM6000 microscope equipped with a Leica DFC350FX camera and then analysed by Fiji/ImageJ software. Four samples for each time point were analysed.

### Immunofluorescence

Samples were fixed with paraformaldehyde 4% (PFA) for 30 min at room temperature (RT). For CD31, βNGF, and DSPP staining, samples were permeabilized by incubating them in 0.2% Tween‐20/PBS (Sigma‐Aldrich, P1379) for 10 min, and then nonspecific sites were blocked by incubating the chambers in PBS supplemented with 0.2% Tween‐20 and 3 mg mL^−1^ bovine serum albumin (BSA; Roth, 1ET5.4) for 1 h at room temperature. HUVECs, tgg, and hDPSCs‐derived odontoblast‐like cells samples on the chip were incubated overnight at 4 ^°^C with the following primary antibodies: mouse anti‐CD31 (Abcam, ab9498) and rabbit anti‐DSPP (Kerafast, ENH081‐FP), all with a 1:100 dilution. For Ki‐67 immunostaining (1:100, Abcam, ab16667), fixed samples were permeabilized with 0.1% triton‐X in PBS and nonspecific sites were blocked as previously described. Samples were washed four times with PBS and incubated overnight with the following secondary antibodies: Alexa‐fluor 568 conjugated anti‐mouse, Alexa‐fluor 488 conjugated anti‐rabbit, Alexa‐fluor 647 conjugated anti‐goat, Alexa‐fluor 647 conjugated anti‐rabbit (1:500; ThermoFisher). Cells were then incubated for 1 h with cytoskeletal and nuclear counterstaining solutions, namely PBS supplemented with 1 mg mL^−1^ BSA, 10 U mL^−1^ Alexa Fluor 488 phalloidin (ThermoFisher, A12379), and 10 µg mL^−1^ DAPI (Sigma‐Aldrich, D9542). Samples were imaged with a Leica DM6000 microscope equipped with a Leica DFC350FX camera and the Leica Application Suite Advanced Fluorescence (LAS AF) software. Confocal imaging was performed with a Zeiss LSM 980 Airyscan confocal microscope. Final images were obtained by Fiji^[^ ^106^ ^]^ and Imaris (Bitplane) software.

### Vascularization Analysis

Fiji and AngioTool software^[^ ^110^ ^]^ were adopted to characterize the endothelial cell network. In particular, the area of vascularization, vessel length, junction density, and the number of connections were analyzed between the adjacent channels.

### RNA Isolation and RT‐qPCR

Cells cultured for 7 days on the chip were lysed with TRIzol Reagent (ThermoFisher, 15596026). For each experiment, cells derived from 7 chips were pooled together. RNA was extracted by exploiting the chloroform/isopropanol phase separation method. Sample purity and RNA concentration were measured with a NanoDrop ND‐1000 UV–vis spectrophotometer (ThermoFisher). Reverse transcription of the isolated RNA was performed using the iScript cDNA synthesis kit according to the manufacturer's instructions (Bio‐Rad, 1708890). cDNA samples were diluted to a final concentration of 10 ng µL^−1^ and stored at ‐80 °C until use. Quantitative polymerase chain reaction (qPCR) was performed by SYBR Green technology. Reactions were set up for 10 ng of cDNA in a final volume of 10 µL in a 96‐well plate (Bio‐Rad, HSP9601) and processed in a CFX Real‐Time PCR Detection System (Bio‐Rad). PCR master mix contained 5 µL Power SYBR Green PCR Master Mix (Biorad, 1725150), 10 µm of reverse and forward primers (Microsynth), and nuclease‐free water was added up to 20 µl final volume. Primers were designed by primer‐BLAST (NCBI) within the sequences of a panel of genes specific for stemness maintenance (*NANOG*, *CD90*, *BMI1*) and odontoblast differentiation (*OSX*, *RUNX2*, *DMP1*) on an exon‐exon junction to prevent genomic DNA amplification (**Table** **1**). The cycling conditions were 95 °C for 10 min, followed by 40 cycles with 95 °C for 15 s, 55 °C for 30 s, and 60 °C for 1 min. Expression levels were normalized to the Ct‐value of the GAPDH housekeeping gene, and the Delta Ct was represented in the graph.

**Table 1:** Forward and reverse sequences of human oligonucleotide primers for RT‐qPCR analysis.

RT‐qPCR primers			
Housekeeping	**GAPDH**	Fw	AGGGCTGCTTTTAACTCTGGT
		Rv	CCCCACTTGATTTTGGAGGGA
Stemness	**NANOG**	Fw	CAACTGGCCGAAGAATAGCAATG
		Rv	TGGTTGCTCCAGGTTGAATTGTT
	**CD90**	Fw	AATACCAGCAGTTCACCCAT
		Rv	GCTAGTGAAGGCGGATAAGT
	**BMI1**	Fw	CCAGGGCTTTTCAAAAATGA
		Rv	CCGATCCAATCTGTTCTGGT
Odontogenesis	**OSX**	Fw	CCTCTGCGGGACTCAACAAC
		Rv	AGCCCATTAGTGCTTGTAAAGG
	**RUNX2**	Fw	CCGCTTCAGTGATTTAGGGC
		Rv	GGGTCTGTAATCTGACTCTGTCC
	**DMP1**	Fw	GTGCCAAGACAGAGAGCTAT
		Rv	AACCCTATGCAACCTTCCAA
	**ALP**	Fw	GCTGTAAGGACATCGCCTACCA
		Rv	CCTGGCTTTCTCGTCACTCTCA

### Statistical Analysis

Data were expressed as means ± standard deviations (N≥3). Statistical analyses were performed using an unpaired t‐test and analysis of variance (ANOVA) to assess significant differences among two or more groups, respectively.

### Ethical Approval Statement

The procedure for anonymized human teeth collection at the Centre of Dental Medicine, Zurich, was approved by the Kantonale Ethikkommission of Zurich, and the patients gave their written informed consent (reference number 2012‐0588). All mice were maintained and handled according to the Swiss Animal Welfare Law and in compliance with the regulations of the Cantonal Veterinary Office, Zurich (ethical approval: ZH205/2020).

## Conflict of Interest

The authors declare no conflict of interest.

## Author Contributions

A.C. and D.S. contributed equally to this work and share first authorship. T.A.M. performed conceptualisation. A.C., D.S., P.P., M.R., and T.A.M. performed methodology. A.C., D.S., P.P., M.R., and T.A.M. performed data analysis. A.C., D.S., P.P., B.S., R.V., M.R., and T.A.M. performed validation. A.C., D.S., P.P., M.R., and T.A.M. performed formal analysis. A.C. and D.S. performed investigation. M.R. and T.A.M. managed resources and reagents. A.C., D.S., P.P., R.V., M.R., and T.A.M. performed data curation. A.C. and D.S. wrote original draft. A.C., D.S., P.P., B.S., R.V., M.R., and T.A.M. wrote, reviewed, and edited. A.C., D.S., P.P., B.S., R.V., M.R., and T.A.M. performed visualisation. T.A.M. performed supervision and project administration. T.A.M. and M.R. performed funding acquisition.

## Supporting information



Supporting Information

Supplemental Video 1

Supplemental Video 2

## Data Availability

The data that support the findings of this study are available from the corresponding author upon reasonable request.
