# Supplementary material for: An Innovative “Tooth‐On‐Chip” Microfluidic Device Emulating the Structure and Physiology of the Dental Pulp Tissue
Source: Adv Healthc Mater. 2025 Aug 21;15(1):e02080. doi: 10.1002/adhm.202502080 (PMC12790315; doi:10.1002/adhm.202502080)
Supplement: Supplementary file 1 — Supporting Information [file ADHM-15-0-s002.docx]

**Supplementary Materials**

**Supplementary Figure 1**

**
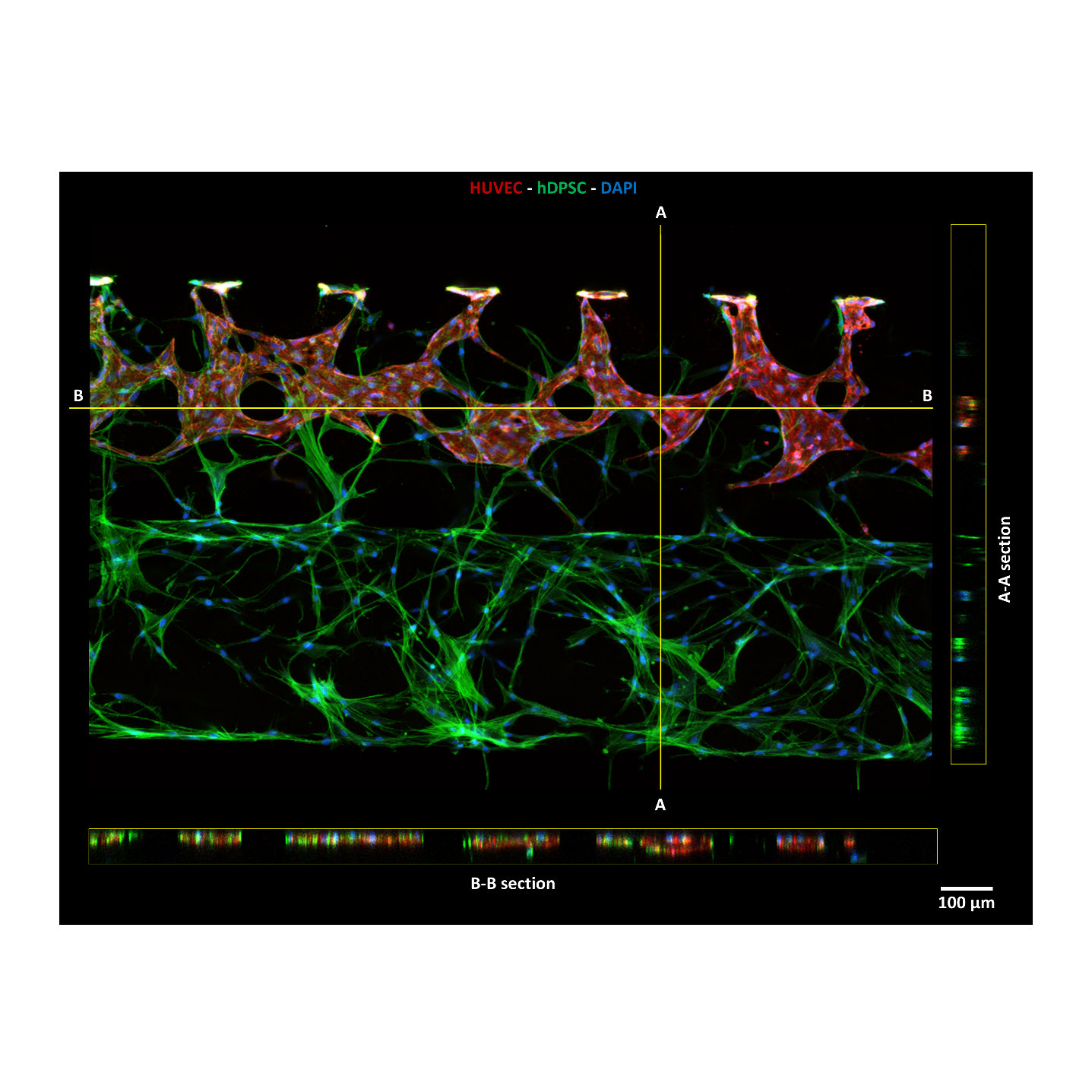
**

**Supplementary Figure S1. Vascularised pulp compartment.** Additional confocal microscopy images of stained HUVECs and hDPSCs at day 4 with CD31 (red) and phalloidin (green) for F-actin, respectively. Nuclei are visualised with DAPI (blue). Orthogonal views (A-A sections and B-B sections) show the lumen of the vascular 3D network.

**Supplementary Material 1. “Tooth-on-chip” 3D model 1 (Video).** 3D confocal image obtained by immunofluorescence microscopy of the innervated and vascularised pulp compartments: HUVECs (red), hDPSCs (green), and tgg (purple), all counterstained with DAPI (blue).

**Supplementary Material 2. “Tooth-on-chip” 3D model 2 (Video)**. 3D confocal image obtained by immunofluorescence microscopy of the dentine-odontoblasts interface and the pulp compartment: HUVECs (red), hDPSCs (green), functional hDPSCs-derived odontoblast-like cells (light blue), and dentine (light blue).
